# A pan-cancer analysis of molecular characteristics and oncogenic role of gasdermins

**DOI:** 10.1186/s12935-022-02483-4

**Published:** 2022-02-14

**Authors:** Mingchao Mu, Qiaoling Yu, Qin Zhang, Jing Guo, Xingjie Wang, Xuejun Sun, Junhui Yu

**Affiliations:** 1 Department of General Surgery, The First Affiliated Hospital of Xi’an Jiaotong University, Xi’an, 710061 Shaanxi China; 2 Department of Pathology, The Second Affiliated Hospital of Xi’an Jiaotong University, Xi’an, 710004 Shaanxi China; 3 Department of Dermatology, The Second Affiliated Hospital of Xi’an Jiaotong University, Xi’an, 710004 Shaanxi China

**Keywords:** Gasdermins, Pyroptosis, Pore-forming protein, Pan-cancer, Drug resistance, Angiogenesis

## Abstract

**Background:**

The gasdermins (GSDMs) family is proposed to be pore-forming effector proteins that cause cell membrane permeabilization and pyroptosis. Despite our increasing knowledge of GSDMD, GSDME and GSDMB, the biological functions and the regulation of GSDM expression and activation remain elusive for most GSDMs. In this study, we analyzed the molecular characteristics and oncogenic role of GSDM family genes systematically.

**Methods:**

TCGA, CCLE, cBioPortal, GEPIA, CellMiner and BioGRID databases were utilized in this study. Immunohistochemical analysis and a series of in vitro experiments were conducted.

**Results:**

We found that, in cancer, GSDM genes and their expressions extensively changed, which were associated with patient survival. The expression of GSDMs was widely associated with cancer-related pathways, drug resistance, immune subtypes, tumor microenvironment and cancer cell stemness. However, an intra- and inter-cancer heterogeneity was discovered regarding the corresponding GSDM gene. We found that GSDMA and GSDMB regulated drug resistance to the opposite direction of GSDME. In colorectal cancer, GSDME might be a positive regulator in cell invasion and metastasis through cell migration and angiogenesis, while GSDMA, GSDMB and GSDMD might be a negatively regulator of cell migration.

**Conclusions:**

GSDM family genes might play important roles in cancer other than pyroptosis. We suggest more efforts be made to investigate the GSDM family and each GSDM gene be studied as an entity in each type of cancer.

**Supplementary Information:**

The online version contains supplementary material available at 10.1186/s12935-022-02483-4.

## Background

The gasdermins family are proposed to be pore-forming effector proteins that cause membrane permeabilization and pyroptosis [1]. The name gasdermin (gas-dermin) is originally based on the relative unique expression in the upper gastrointestinal tract and dermis [2]. In humans, the GSDM family contains six members, including GSDMA, GSDMB, GSDMC, GSDMD, GSDME (a.k.a. DFNA5) and PJVK (a.k.a. DFNB59) (Additional file 1: Fig. S1). In mice, homolog of GSDMB is absent but three forms of GSDMA, GSDMA1-3, and four homologs of GSDMC, GSDMC1-4, are present. All GSDM proteins but PJVK share a similar architecture consisting of a functionally important N-terminal pore-forming domain (GSDM-N), a regulatory C-terminal domain (GSDM-C) and a linker between them. The full-length protein before cleavage is considered to be inactive in inducing pyroptosis. In this autoinhibited state, the C-terminal domain binding to the N-terminal effector domain to repress its activity [3–7]. In vitro, actually, the gasdermin-N and -C domains remained bound together following cleavage at inter-domain linker (Additional file 2: Fig. S2) [8]. A cleavage within the linker region and consequent separation of the two domains is considered to be prerequisite for activation [5, 9–11]. Once cleaved, the cytotoxic N-terminal fragment is liberated and free to form membrane pores through oligomerization and insertion into the plasma membrane, resulting in loss of osmotic homeostasis, cell swelling, and death.

Despite our increasing knowledge of GSDMD, GSDME and GSDMB, the biological functions and the regulation of GSDM expression and activation remain elusive for most GSDMs. A more comprehensive investigation into the function and mechanism of the GSDM family will be beneficial to elucidate GSDM-mediated diseases and consequently development of GSDM-targeting therapeutics.

## Methods

### Phylogenetic relationship, gene structure, conserved motif analysis

Evolutionary analyses were conducted in MEGA7 [12]. Gene structure was analyzed by GSDS 2.0 [13]. Conserved motifs were identified using the online tool MEME 5.3.3 [14].

### Genomic alteration analysis

Analysis of genomic alteration of GSDM members in pan-cancer was performed by Cbioportal (http://www.cbioportal.org/) [15, 16]. All types of TCGA Pan-Cancer Atlas available (10,967 samples) were selected for calculation. Mutation including missense, truncating, in frame and fusion gene were examined in the coding sequence of each gene.

### Gene expression and survival analysis

TCGA pan-cancer data RNA-Seq (RNA SeqV2 RSEM) and patient survival data were download from Xena browser (https://xenabrowser.net/datapages/). 33 types of cancer data were obtained, involving ACC, BLCA, BRCA, CESC, CHOL, COAD, DLBC, ESCA, GBM, HNSC, KICH, KIRC, KIRP, LAML, LGG, LIHC, LUAD, LUSC, MESO, OV, PAAD, PCPG, PRAD, READ, SARC, SKCM, STAD, TGCT, THCA, THYM, UCEC, UCS and UVM. Cancer types with no more than 5 normal adjacent tissue as control (15 cancer types) was excluded when analyze the differential expression. Genetical regulation of GSDM gene expression was analyzed by Cbioportal(http://www.cbioportal.org/) [15, 16].

### Methylation and cancer-related pathways analysis

The differential methylation and cancer-related pathways were analyzed by GSCALite (http://bioinfo.life.hust.edu.cn/web/GSCALite/) [17]. All six GSDM members and 33 types TCGA cancer data were selected for analysis.

### Drug resistance analysis

The NCI-60 data was accessed from the CellMiner 2.4.2 [18] (https://discover.nci.nih.gov/cellminer/). The drug without FDA approval were excluded.

### Protein–protein interaction analysis

The proteins interacting with GSDM genes were searched from BioGRID 4.3.195 (https://thebiogrid.org/), followed by visualization by Cytoscape 3.8.1.

### Tumor microenvironment analysis

The estimated score was generated by the ssGSEA algorithm and the R script followed as estimateScore (input.ds = "commonGenes.gct", output.ds = "estimateScore.gct").

### Tumor stemness analysis

Stemness scores based on mRNA (RNAss) and DNA-methylation (DNAss) were downloaded from xena browser (https://xenabrowser.net/datapages/).

### Immunohistochemistry

A total of 16 human CRC tissue were obtained from patients who had undergone open surgery for cancer resection at the First Affiliated Hospital of Xi'an Jiaotong University (Xi'an, China) between Dec. 2020 and Jan. 2021. Tissues were then formalin-fixed, embedded in paraffin wax and sectioned into 4 μm thick sections. The sections were incubated with primary antibodies against GSDME (1:200, ab215191, Abcam), CD34 (1:800, 14,486-1-AP, Proteintech) overnight at 4˚C, followed by HRP-conjugated secondary goat antibody (ZSGB-Bio). The staining intensity was divided into scores 0, 1, 2 and 3 representing none, weak, moderate, and strong, respectively. The percentage of positive cells was separated into < 5%, 5–25%, 25–50%, 50–75% and ≥ 75% representing 0, 1, 2, 3 and 4 respectively. Immunoreactivity score (IRS) was generated by multiplying the staining intensity score by the percentage of positive cells score. GSDME IRS < 6 was considered as low expression of GSDME, while IRS ≥ 6 was considered as high expression of GSDME.

### Cell cultures and lentiviral vectors and transfection

Colon cancer cells HCT116 and SW480 (Shanghai Institute of Cell Biology, Chinese Academy of Sciences) were all routinely cultured in Dulbecco’s modified Eagle’s medium (DMEM) (BI-USA) supplemented with 9% fetal bovine serum (FBS)(LONSERA) at 5% CO_2_ at 37 °C. The phU6-EGFP-GSDME lentiviral vector and its control vector was constructed and prepared by GeneChem Co., Ltd. All transfections were performed according to the manufacturer's instructions.

### Western blot assay

Cells were lysed with RIPA buffer (Beyotime) containing protease inhibitor. Cell lysates were heat denatured, separated by SDS-PAGE, transferred to PVDF membranes (Invitrogen), blocked with 5% skimmed milk. Then membranes were incubated respectively with primary antibodies: GSDME (1:500, ab215191, Abcam), GAPDH (1:5000, 10494-1-AP, Proteintech) overnight at 4℃ and secondary antibodies, goat anti-rabbit IgG (1:5000, bs-0295G, Bioss) for 1 h at room temperature. Immunoblots were visualized using an ECL detection reagent (Minipore).

### Wound-healing assays

HCT116 and SW480 cells were cultured in 6-well plates until 95–100%confluent. Then the monolayers were scratched using pipette tips (10 μL). After scratching, cell was washed and maintained in serum-free medium for 72 h. Each experiment was performed in triplicate.

### Transwell assays

Cell migration and invasion were measured using Boyden chambers in 24-transwell plates (8 μm pores, Corning). In both assays, the lower chambers were filled with 600 μL DMEM medium containing 10% FBS. In the migration assay, 2.5 × 10^4^ cells were seeded in upper chamber. In invasion assay, 5 × 10^4^ cells were seeded in upper chamber pre-coated with 60 μL of Matrigel (BD). The cells were incubated at 37 °C, 5% CO_2_ for 48 h. After incubation, non-migratory cells were removed by cotton swab. Then the membranes were fixed with 4% paraformaldehyde and stained with 1% crystal violet. The number of cells was counted in five randomly selected fields. Each experiment was performed in triplicate.

### Statistics analysis

The Wilcoxon Signed-rank Test was used for analyzing the differential expression of GSDM gene between tumor and normal tissue. Kruskal–Wallis rank sum test was used for comparing GSDM gene expression among immune subtypes. Pearson's correlation tests were used for analyzing the correlation among GSDM gene members and the correlation between GSDM gene expression and z scores for cell sensitivity data. Spearman's Rank-Order Correlation was used for analyzing association between gene expression and Estimate/Stemness scores. The Kaplan–Meier method with log-rank test were used for comparing survival. Cox regression was used for analyzing risk factors on survival. P value < 0.05 indicated statistical significance. Besides online analysis, all statistics were calculated by R Project (Version 4.0.3).

## Results

### Extensive genomic alterations of GSDM genes in cancer

Genomic alterations were defined as gene amplifications, deep deletions, fusion, truncating mutations, in-frame mutations, missense mutations or splice mutation using the cBioPortal definitions [15, 16]. Copy number alterations (CNAs) were classified as gene amplifications, gain, shallow deletion and deep deletions. We performed mutation and CNAs analysis of GSDM genes to identify genomic alterations across the Pan-Cancer cohort.

**Fig. 1:**
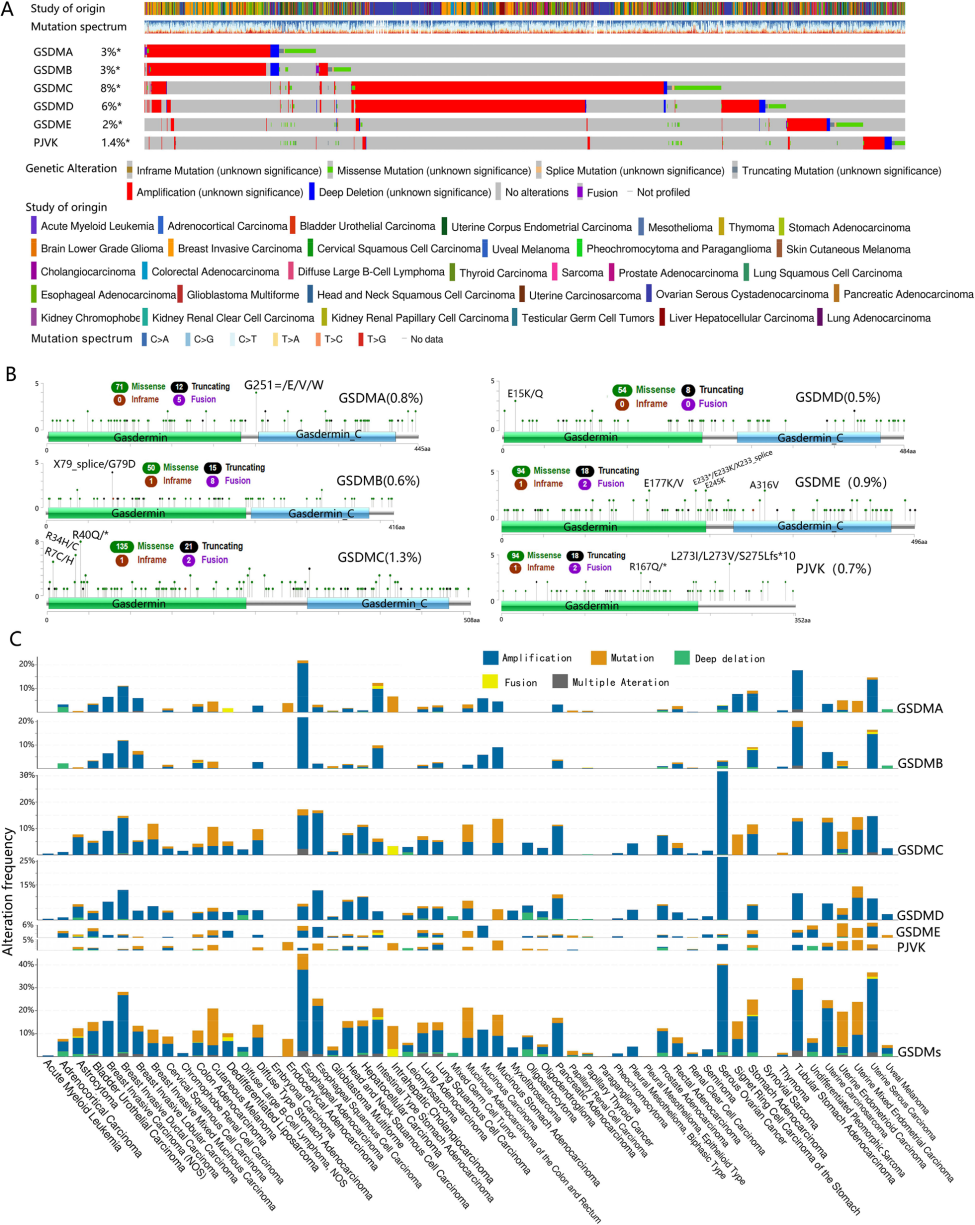
Alteration of GSDM family genes. **A** An OncoPrint showing GSDM genetic alterations across TCGA cancer studies. Each sample was represented as a column and each GSDM gene was represented as a row. The alterations were represented in different colors. **B** Representation of the mutations along GSDM genes. Green dots represent missense mutations, black dots represent truncating mutations, brown dots represent in-frame mutations and purple dots represent gene fusions. **C** Alteration frequency, mutation, copy number alteration (CNA) and gene fusion of GSDM genes across cancer types

Oncoprint representation from cBioPortal revealed the distribution of GSDM genomic alterations in the Pan-Cancer (Fig. 1A). As a result, GSDM family genes were altered in 1588 (14%) of 10,953 patients in 32 TCGA studies, and the overall alteration frequency of each gene in the GSDM family was 1.4–8%. GSDMC (8%) and GSDMD (6%) had the overall higher gene alteration frequency than GSDMA (3%), GSDMB (3%), GSDME (2%) and PJVK (1.4%). CNAs played a predominant role in gene alteration frequency in GSDMD, GSDMA, GSDMB and GSDMC, while it played an approximate equal role as mutation in GSDME. Noticeably, PJVK had more mutations other than CNAs, setting it apart.

The overall average somatic mutation frequency of each gene in the GSDM family was 0.5–1.3% (Fig. 1B). The majority of mutations were almost distributed evenly in the coding sequence of GSDM genes. The most common mutations of GSDM genes were missense and truncating mutations. GSDMC (1.3%) had the highest mutation rate among the six members, while GSDMD (0.5%) had the lowest. In GSDMC, there were 135 Missense, 21 Truncating and 7 splice mutations and the hotspot mutations (R40Q/*, R34H/C, R7C/H) were all located within the Gerdermin domain. In GSDMD, there were 54 Missense, 8 Truncating and 2 splice mutations and the hotspot mutations (E15K/Q) were also located within the Gerdermin domain.

Of the 57 cancer types, 53(93%) had genomic alterations in GSDM family genes (Fig. 1C). Genomic alteration frequencies varied widely across cancer types, from no alterations in embryonal carcinoma, synovial sarcoma, paraganglioma and pleural mesothelioma (epithelioid), to 44.83% alteration frequency in esophageal adenocarcinoma. Genomic alteration type also varied greatly across tumor types, from no alterations or single type in acute myeloid leukemia, pheochromocytoma, chromophobe renal cell carcinoma, mixed germ cell tumor, seminoma, myxofibrosarcoma, pleural mesothelioma (biphasic) and endocervical adenocarcinoma, to multiple type alteration in most other cancers. Among the 57 types, esophageal adenocarcinoma (44.83%), serous ovarian cancer (40.41%) and uterine serous carcinoma (36.7%) had the highest frequency of GSDM genes alteration, while chromophobe renal cell carcinoma (1.54%), pheochromocytoma (1.36%) and acute myeloid leukemia (0.5%) had the lowest. Serous ovarian cancer had the highest amplification frequency (38.01%). Cutaneous melanoma had the highest GSDM genes mutation frequency (15.99%). GSDMA mutation was more likely associated with intrahepatic cholangiocarcinoma, uterine mixed endometrial carcinoma and cutaneous melanoma. GSDMB mutation was more likely associated with cutaneous melanoma. GSDMC and GSDMD mutations were more likely associated with mucinous stomach adenocarcinoma. GSDME and PJVK mutation were related to uterine endometrioid carcinoma and uterine mixed endometrial carcinoma, respectively.

In most cancer types, amplification was the major contributor to the GSDM gene alteration (Fig. 1C). Esophageal adenocarcinoma had the highest frequency of GSDMA (20.69%) and GSDMB (21.84%) amplification. Serous ovarian cancer had the highest frequency of GSDMC (31.51%) and GSDMD (26.71%) amplification. Mucinous carcinoma and serous ovarian cancer had the highest frequency of GSDME (5.88%) and PJVK (2.91%) amplification, respectively. Intrahepatic cholangiocarcinoma, cutaneous melanoma, uterine endometrioid carcinoma and uterine mixed endometrial carcinoma had the highest GSDMA (6.67%), GSDMB (2.7%), GSDME (6.52%) and PJVK (4.76%) mutation frequency, respectively. Mucinous stomach adenocarcinoma had the highest GSDMC (9.09%) and GSDMD (4.55%) mutation frequency.

### Expression of GSDM genes extensively changed in cancer

**Fig. 2:**
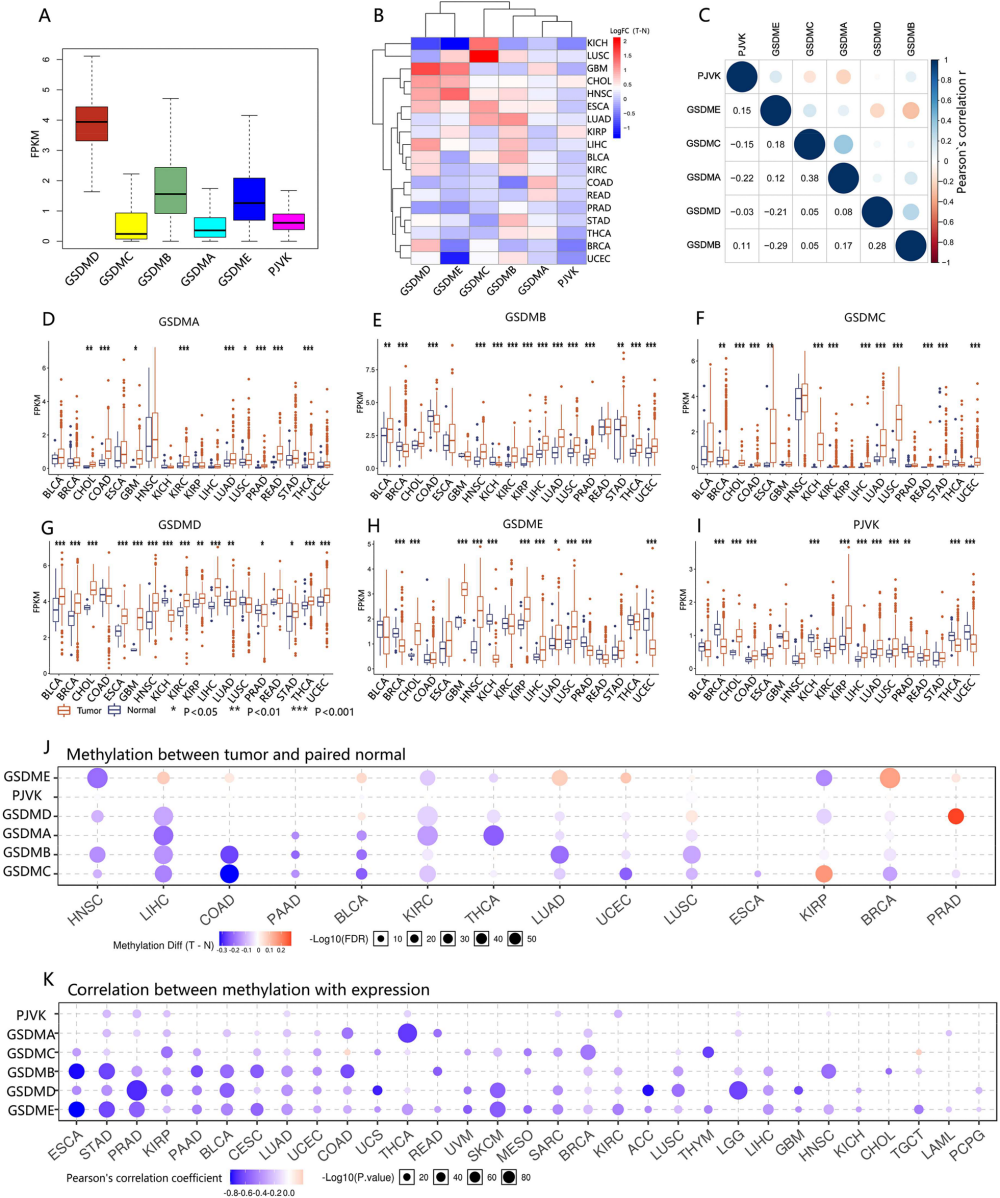
The expression of GSDM family genes. **A** Box plots represent the relative expression of GSDM genes in pan-cancer. **B** Heatmap showing differential expression of GSDM genes across cancer types. **C** Matrix graph of Pearson’s correlation of GSDM genes expression in pan-cancer. **D**–**I** Box plots represented the differential expression of six GSDM genes between tumor and normal tissue. **J** Methylation difference between tumor and paired normal tissue. P value was adjusted by FDR, FDR <  = 0.05 was considered as significant. Only significant results were shown as bubble plots. **K** Pearson’s Product-moment Correlation between GSDM genes expression and corresponding gene methylation. P-value was adjusted by FDR and genes with FDR <  = 0.05 will be showed

We explored the expression levels of the six GSDM family members in all 33 cancer types available in TCGA pan-cancer data. In pan-cancer, GSDMD had the relatively highest expression level while GSDMC had the lowest (Fig. 2A). Overall, GSDM family genes tended to be upregulated compared with their adjacent normal tissue (Fig. 2B, D–I). However, a noticeable intra- and inter-cancer heterogeneity regarding the expression levels of the corresponding genes was unveiled for all six GSDM members. There was no intrinsic unified pattern in every single GSDM gene expression. In addition, expression levels of GSDM family members were significantly (P <  = 0.001) correlated with each other in pan-cancer based on Pearson's correlation tests (Fig. 2C). However, the absolute value of the correlation coefficient ranged from 0.03 to 0.38, testifying a weak or negligible correlation. These findings revealed the intrinsic differences in the expression of GSDM genes between different cancer types or between different GSDM family members. Whether a specific GSDM gene was an oncogene or an anti-oncogene cannot be decided without choosing a cancer type. The complexity of expression spectrum necessitated a further study of each individual GSDM gene member as an entity.

Genetically, the copy number aberrations (CNAs) might be a considerable molecular mechanism leading to the disturbance of gene expression of GSDM family (Additional file 3: Fig. S3). Overall, the expression level of GSDMs was upregulated in gain and amplification but downregulated in shallow deletion and deep deletions in pan-cancer. Regarding the widely amplifications and relatively few deep deletions in GSDM genes, the overall upregulated expression of corresponding gene seemed to be reasoned.

Epigenetically, we identified the effects of promoter methylation on GSDM family gene expression in 33 types of cancer by integrating methylation levels and expression profile data (Fig. 2J). 14 (42.4%) of 33 types of cancer were significantly (FDR <  = 0.05) differential methylated in GSDM gene promoters. Overall, GSDM promoters were hypomethylated in variety of cancer and the expression of GSDM genes was negatively correlated with promoter methylation level (Fig. 2K). This finding corresponded to the overall up-regulated GSDM genes expression.

### GSDM genes involving widely in cancer-related pathways and drugs sensitivity

**Fig. 3:**
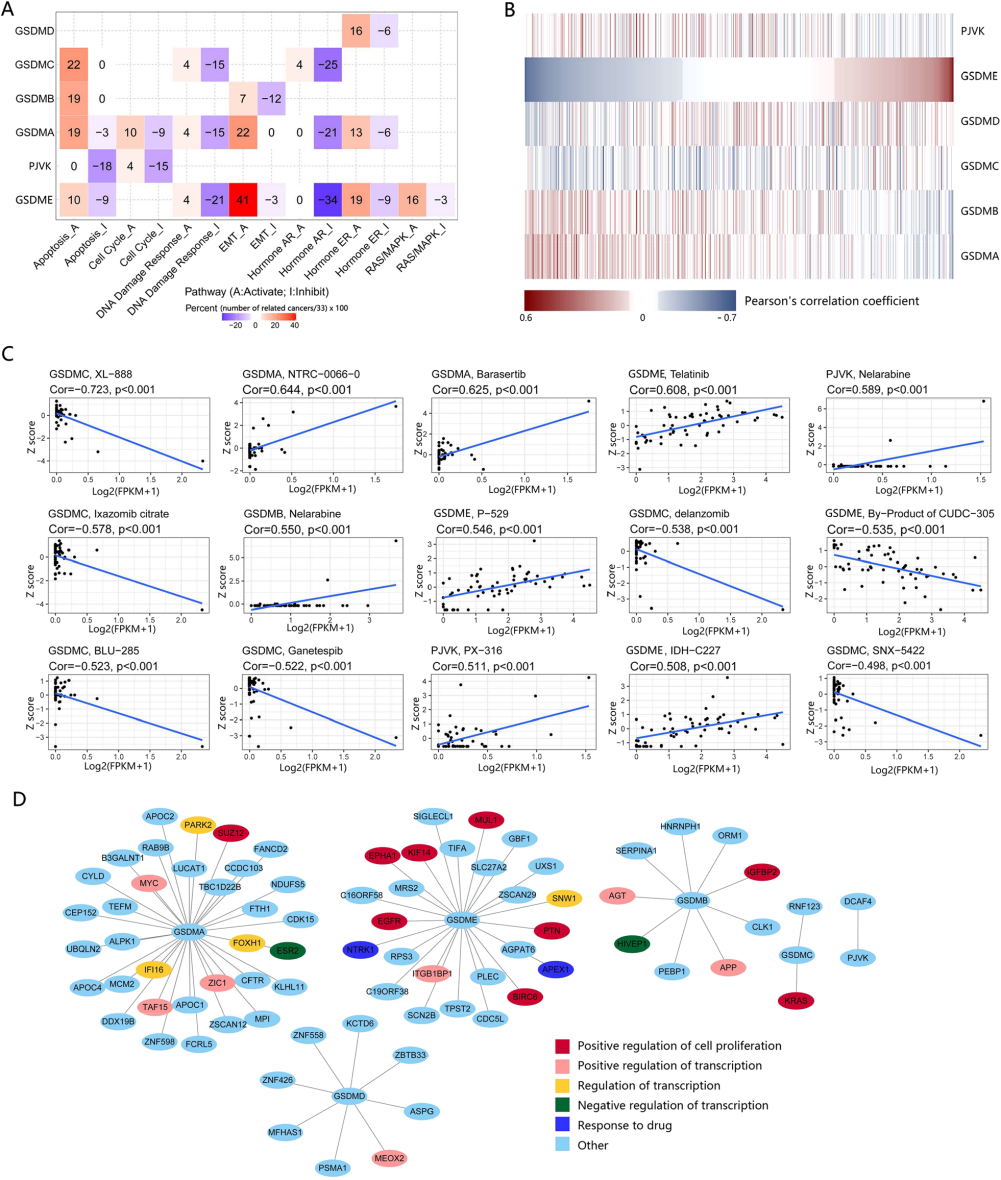
GSDM genes expression involving widely in cancer-related pathways and drug resistance. **A** Heatmap showing correlation between GSDM genes expression and 10 cancer related pathways. PI3K/AKT, TSC/mTOR and RTK were excluded for they significantly (FDR <  = 0.05) correlated with GSDM genes in no more than 4 of 33 cancer types. Each pathway (activate or inhibit) was represented as a column and each GSDM gene was represented as a row. **B** Heatmap showing correlation between expression of GSDM genes and drug sensitivity. Each drug was represented as a column and each GSDM gene was represented as a row. Notice that Pearson's correlation coefficient was ranked by GSDME. **C** Scatter Plot showing the correlation between GSDM genes expression (in horizontal axis) and drug sensitivity (in vertical axis). Only the 15 with the smallest P values were shown. **D** Protein–protein interaction (PPI) network analysis of GSDM genes. The interaction was downloaded from BioGRID and visualized by Cytoscape software

We investigated the correlation between the expression of GSDM genes and ten famous cancer related pathways, including TSC/mTOR, RTK, RAS/MAPK, PI3K/AKT, Hormone ER, Hormone AR, EMT, DNA Damage Response, Cell Cycle and Apoptosis pathways. As a result, the expression of GSDM family genes was correlated with the activation or inhibition of a variety of cancer related pathways (Fig. 3A). However, the activating or inhibiting role on the cancer related pathways of GSDM genes varied depending on types of cancer, also, types of pathways. Noticeably, the high expression of GSDME was significantly (FDR <  = 0.05) correlated with EMT activation in 41% of cancer types.

We further investigated the correlation between drug sensitivity and GSDM family genes expression level in CellMiner [18]. We found that the expression level of GSDM family genes was significantly correlated (P < 0.05) with drug sensitivity in 309 (41.3%) of 748 (under FDA approved or clinical trial) chemical compounds and natural products (Fig. 3C, Additional file 8: Data S1). GSDMA, GSDMB, GSDMC, GSDMD, GSDME and PJVK were correlated with sensitivity of 34, 31, 49, 39, 88 and 37 drugs, respectively. Obviously, the expression of GSDME was more likely to be associated with drug sensitivity. The extent or the direction of the correlation was not unified regarding every GSDM gene and every drug. For example, the high expression of GSDME increased drug sensitivity of Telatinib, P–529 and IDH–C227, but increased drug resistance of Vinorelbine and Des-fluoro-TAK-960. Interestingly, GSDMA and GSDMB generally went to the same direction of correlation when associated with drug sensitivity of a specific drug (Fig. 3B, Additional file 9: Data S2), but the opposite direction of GSDME. For example, the drug sensitivity of PI-103 was negatively correlated with the expression of GSDMA (r = − 0.35) and GSDMB (− 0.43), but it was positively correlated with the expression of GSDME (r = 0.31). The drug sensitivity of AM-5992 negatively correlated with GSDME (r = − 0.41), but it positively correlated with the expression of GSDMA (r = 0.39) and GSDMB (r = 0.36). These results implied the possibility that GSDMA and GSDMB were involved in some pathways together with GSDME, but they played a different role from GSDME. In addition, GSDMC exhibited a great difference in spectrum of correlation with drug sensitivity from the rest of the GSDM family members, indicating a different role GSDMC might play in regulation of drug sensitivity.

We next examined the protein–protein-interaction (PPI) network of GSDM family genes. Through the visualized networks, we found no interaction within GSDM genes, but GSDM genes interacted with several proteins known to be involved in regulation of cell proliferation, transcription and drug resistance (Fig. 3D). These findings implied that the GSDM family genes were widely involved in the regulation of biologic behaviors of cancer and reactions to drugs. However, there was a nonnegligible heterogeneity in the specific mechanism across cancer types or GSDM gene types.

### Patient survival was associated with the expression of GSDM genes

**Fig. 4:**
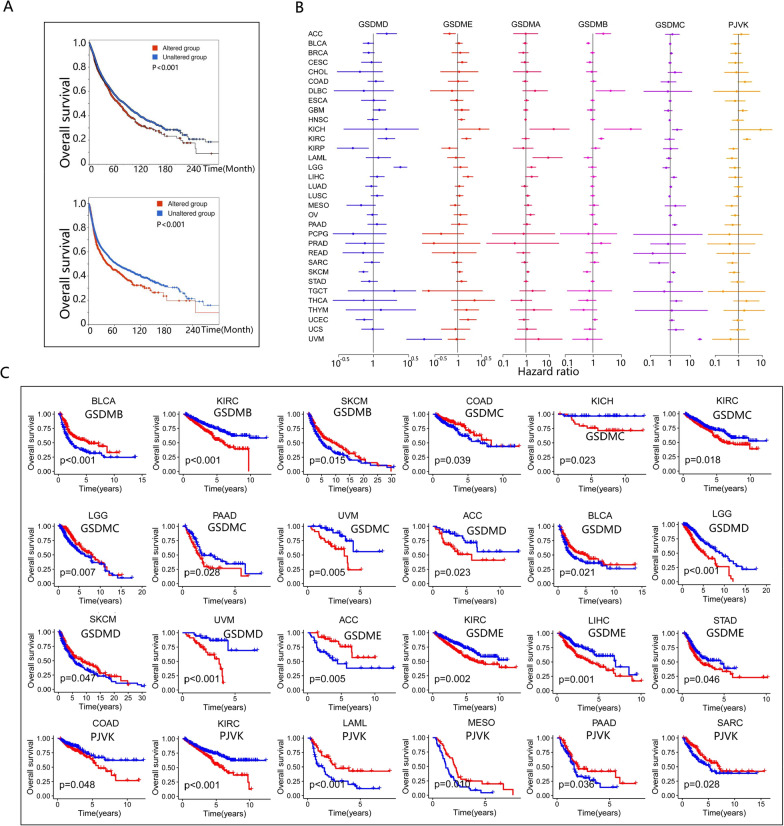
Correlation of GSDM genes expression with patient survival. **A** Kaplan–Meier survival curves for GSDM genes alteration associated with overall survival and progression free survival. GSDM genes altered group associated with poor prognosis in pan-cancer. **B** The forest plots showing the univariate Cox proportional hazards model for correlation between GSDM genes expression and patient survival. **C** Kaplan–Meier survival curves for GSDM genes expression associated with overall survival. Patients were divided into high and low-expression groups defined by expression level of GSDM genes (median was the cutoff)

Since the extensive alteration and differential expression of GSDM genes among cancers, we further investigated the association of GSDM alteration and expression with patient overall survival (OS) and progression free survival (PFS). We found that altered GSDM genes was significant associated with worse OS and PFS in pan-cancer (Fig. 4A). Univariate Cox proportional hazards model demonstrated that the expression level of GSDM genes widely associated with the survival risk (Fig. 4B, Additional file 10: Data S3). However, whether a specific GSDM gene was a high or low survival risk gene varied depending on types of cancer. For instance, high expression of GSDME increased survival risk in KIRC, LIHC and UCEC, but decreased survival risk in ACC. According to KM survival curves (Fig. 4C), OS benefited with high expression of GSDMB in BLCA and SKCM, but with low GSDMB expression in KIRC. High GSDMC expression associated with survival advantage in COAD and LGG, but with survival disadvantage in KIRC, KICH and PAAD. High expression of GSDMD predicted a better OS in BLCA and SKCM, but a worse OS in ACC, LGG and UVM. High expression of PJVK also associated with a better OS in LAML, MESO, PAAD and SARC, but with a worse OS in KIRC and COAD. Interestingly, the expression of GSDMA did not affect OS significantly. In brief, the expression of GSDM genes was extensively involved in patient survival, but it varied by cancer types.

### GSDM genes were associated with immune subtypes and tumor microenvironment (TME)

**Fig. 5:**
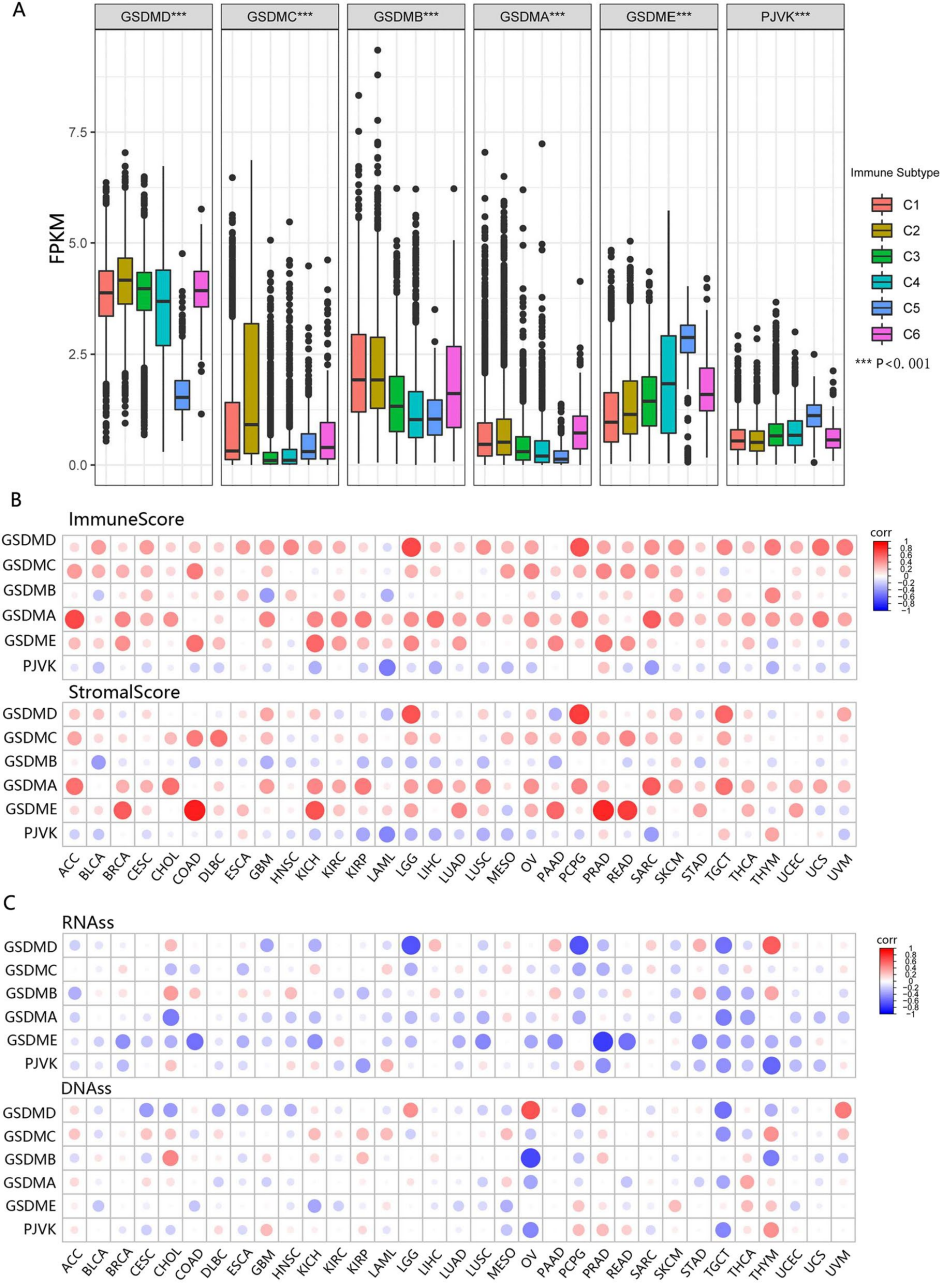
The differential expression of GSDM genes across immune subtypes and the association between GSDM genes expression and TME/DNAss/RNAss. **A** The distribution of GSDM expression across immune subtypes. C1 (wound healing); C2 (IFN-gamma dominant); C3 (inflammatory); C4 (lymphocyte depleted); C6 (TGF-b dominant). **B** Matrix graph of Spearman's Rank-Order Correlation between GSDM genes expression and Immunescore/Stromascore. Cancer types and GSDM genes were shown on the horizontal and vertical axis respectively. **C** Correlation matrix between GSDM genes expression and cancer stemness scores RNAss and DNAss. Cancer types and GSDM genes were shown on the horizontal and vertical axis respectively

Since the GSDM genes were a family of pore-forming proteins implicated in the immune response, we investigated the correlation between GSDM genes and immune infiltrates in cancer to understand how each of the GSDM family members was associated with immune components. A global transcriptomic immune classification of solid tumors had identified six immune subtypes (ISs) (C1-C6) [19]. In pan-cancer, patients classified into type C3 (inflammatory) and C5 (immunologically quiet) associated with significant survival advantage while patients characterized into type C4 (lymphocyte depleted) and C6 (TGFβ dominant) had a significant survival disadvantage (Additional file 4: Fig. S4). Further analysis demonstrated the expression of GSDM family genes was significantly (P < 0.001) correlated with immune subtypes (Fig. 5A). High expressions of GSDMA, GSDMB and GSDMC were associated with subtype C1 (Wound healing), C2 (INF-r dominant) and C6, indicating a cancer promoter role of these three members. High expressions of GSDME and PJVK were associated with subtype C5, indicating these two genes might mainly play a cancer suppressor role. However, GSDMD might play a dual role, since high expression of GSDMD correlated significantly with patients characterized into C3 as well as C4 and C6.

We further investigated the correlation of GSDM genes with ImmuneScore, StromalScore and EstimateScore which estimated the level of immune cell infiltrate, stromal cells infiltrate and tumor purity respectively using the ESTIMATE program [20] (Fig. 5B, Additional file 11: Data S4, Additional file 12: Data S5). The higher ImmuneScore or StromalScore were, the more extensive the immune or stromal components infiltrated in TME. The EstimateScore, negative correlation with tumor purity, was the sum of StromalScore and ImmuneScore. As a result, the expressions of all GSDM genes but PJVK were mainly positively correlated with ImmuneScore. Specifically, GSDMD was strong correlated with LGG (r = 0.68) and PCPG (r = 0.66), and GSDMA was strong correlated with ImmuneScore in ACC (r = 0.69) and SARC (r = 0.60) (Additional file 11: Data S4). PJVK was negatively correlated with ImmuneScore significantly in LAML (r = − 0.5). Regarding StromalScore, the expressions of all GSDM genes but GSDMB and PJVK were mainly positively correlated with StromalScore. To be specific, GSDMD was strong correlated with StromalScore in PCPG (r = 0.76) and LGG (r = 0.64). The most striking was that the expression of GSDME was very strong correlated with StromalScore in COAD (r = 0.84) and PRAD (r = 0.83) and it had a strong correlation in READ (r = 0.72), KICH (r = 0.66) and BRCA (r = 0.60) with StromalScore. In spite of the relatively small coefficient, the expression of GSDMB (|r|< 0.37) and PJVK(|r|< 0.47) were mainly negatively correlated with StromalScore. Not surprisingly, the high expressions of GSDMA, GSDMC, GSDMD and GSDME were observed correlated with low tumor purity (Additional file 5: Fig. S5). Meanwhile, the high expression of PJVK indicated a high tumor purity in most types of cancer. Stromal and immune cells were proposed to play an important role in cancer growth, metastasis and drug resistance [20], indicating GSDM family genes might have a role in regulating tumor behavior by interacting with TME.

### GSDM genes were associated with tumor cell stemness

To evaluate the relationship between GSDM genes and tumor stemness, we investigated the correlation between the expression of GSDM genes and stemness score (RNAss and DNAss). As a result, the association between GSDM genes expression and tumor stemness varied depending on the types of cancer and types of GSDM genes (Fig. 5C, Additional file 13: Data S6). To be specific, RNAss was strongly correlated with GSDMD in LGG (r = -0.66) and PCPG (r = − 0.67), strongly correlated with GSDME in PRAD (r = − 0.75). DNAss was found strongly correlated with GSDMB in OV (r = − 0.71). Besides these negative correlations, GSDMD was found positively associated with RNAss in THYM (r = 0.61), with DNAss in OV (r = 0.64). In spite of the different results estimated by RNAss and DNAss based on their algorithm differences, GSDM genes were found to be correlated with tumor stemness in varying degrees.

### GSDM genes were associated with colorectal cancer cell invasion and metastasis

**Fig. 6:**
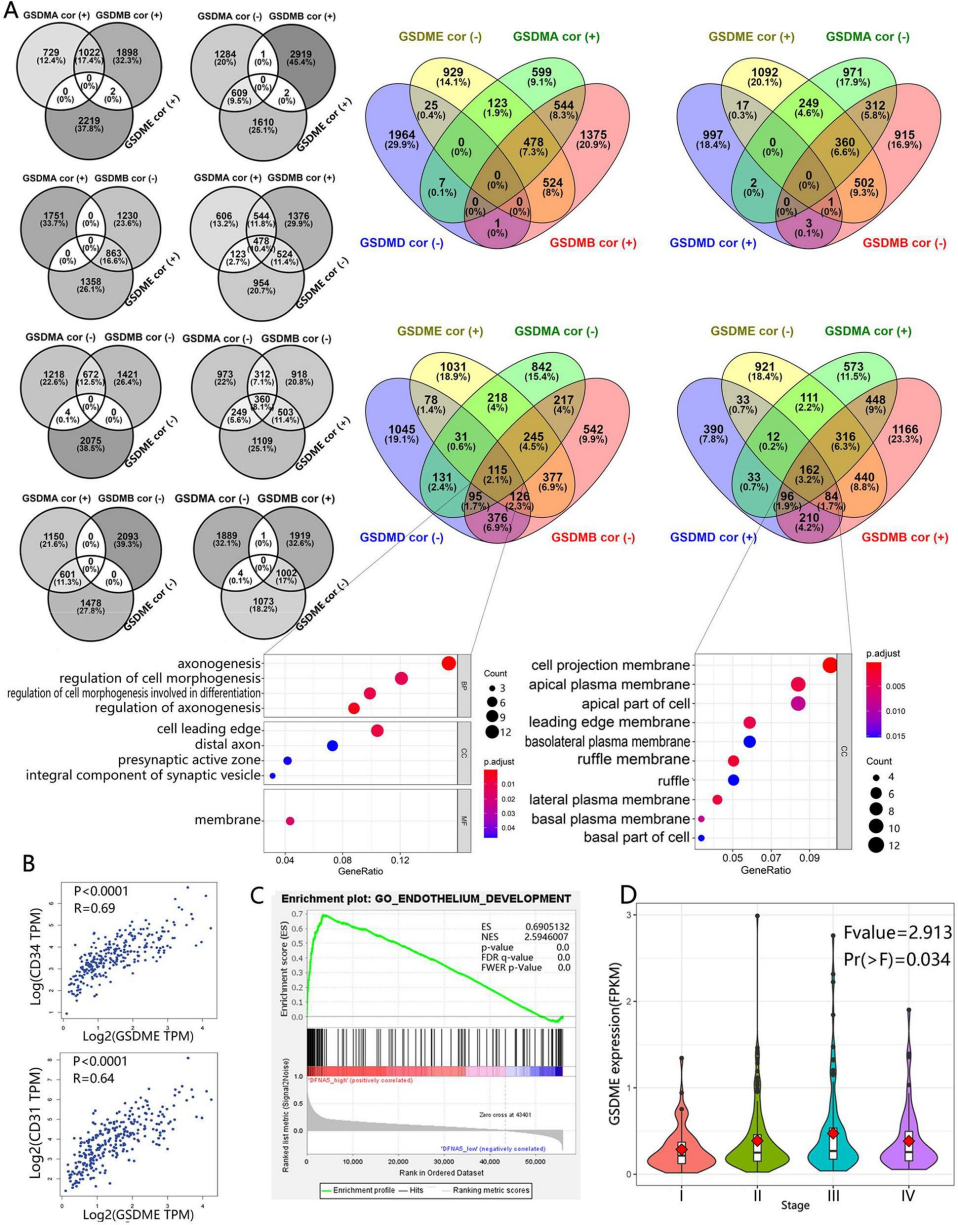
GSDM family was involved in promoting metastasis and angiogenesis of colorectal cancer. **A** These three-circle Venn diagrams showed the interaction between the correlated genes of GSDMA, DSDMB and GSDME. These four-circle Venn diagrams showed the interaction between the correlated genes of GSDMA, DSDMB, GSDME and GSDMD. Notice that there was no or almost no interaction between some groups. The correlated genes of GSDM family members were determined by the Pearson’s Product-moment Correlation test in colorectal cell lines based on Broad Institute Cancer Cell Line Encyclopedia (CCLE) dataset. GSDMA/B/D/E cor (+)/(−) denoted the genes positively/negatively significantly (P < 0.05) correlated with GSDMA/B/D/E. The GO enrichment analysis was performed on the genes correlated with all the four GSDM genes. **B** GSDME expression was positively correlated with the expression of CD31 and CD34 based on TCGA-COAD dataset analysis. **C** Increased expression of GSDME provided endothelial development advantage based on Gene Set Enrichment Analysis (GSEA). **D** Violin plot revealed that GSDME expression was correlated with stage of CRC

Next, we investigated the genes associated (P < 0.05) with GSDM genes in cell lines in CCLE using Venn diagrams [21]. Interestingly, in colorectal cancer cell lines, we found the genes positively correlated with GSDMA had no intersection (none gene) with the genes negatively correlated with GSDMB, but it had intersection (1022 genes) with the genes positively correlated with GSDMB (Fig. 6A). Also, the genes negatively correlated with GSDMA had almost no intersection (only one gene) with the genes positively correlated with GSDMB, but it had intersection (672 genes) with the genes negatively correlated with GSDMB. When it came to GSDME, we found a similar relationship. The genes positively correlated with GSDME had almost no intersection with the genes positively correlated with GSDMA (none gene)/GSDMB (two genes), but it had intersection with the genes negatively correlated with GSDMA (609 genes)/GSDMB (863 genes). In turn, the genes negatively correlated with GSDME had almost no intersection with the genes negatively correlated with GSDMA (four genes)/GSDMB (none gene), but it had intersection with the genes positively correlated with GSDMA (601 genes)/GSDMB (1002 genes). And that, the genes positively correlated with GSDMD had interaction (291 genes) with the genes negatively correlated with GSDME, but had almost no interaction (18 genes) with the genes positively correlated with GSDME. In brief, we suspected that GSDMA, GSDMB, GSDMD and GSDME shared some biofunctions and the direction of regulation of these biofunctions by GSDMA and GSDMB was same to GSDMD but the opposite to GSDME.

We further performed GO enrichment analysis on the GSDMA− GSDMB− GSDMD− GSDME+ gene list (negatively correlated with GSDMA, GSDMB and GSDMD but positively correlated with GSDME) and GSDMA+ GSDMB+ GSDMD+ GSDME− gene list (positively correlated with GSDMA, GSDMB and GSDMD but negatively correlated with GSDME). The GO analysis results revealed that the former list significantly (p-value < 0.05 and q-value < 0.05) enriched in biological processes related to axonogenesis and morphogenesis (shape and size of cancer cells), while the latter gained none enrichment in biological process. Also, the majority of these genes in the two lists were located at the leading edge or projection of cancer cells. Since cell morphological changes were the prerequisite for cell migration and abnormal cell migration underlined invasion and metastasis of cancer cells [22], we suspected that GSDMA, GSDMB, GSDMD and GSDME might be involved in colorectal cancer cell invasion and metastasis, and the former three might played as negative regulators while the last one played as a positive regulator.

**Fig. 7:**
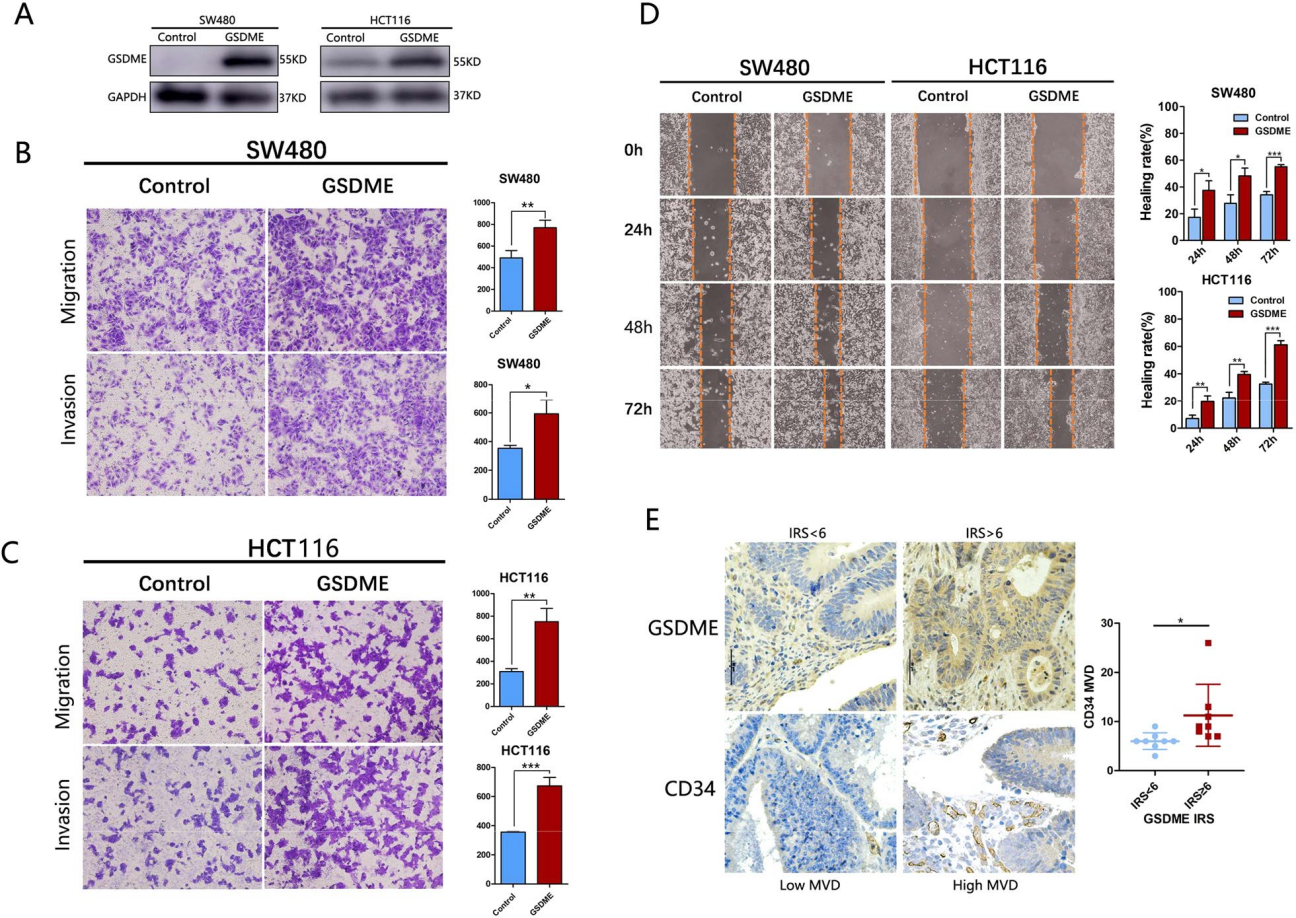
The expression of GSDME was correlated with Micro Vessel density (MVD) and GSDME promoted the invasive and migratory abilities of colon cancer cells in vitro. **A** The GSDME was overexpressed in HCT116 and SW480 cells. **B** Transwell assays in control and GSDME-overexpression SW480 cells. **C** Transwell assays in control and GSDME-overexpression HCT116 cells. **D** Migration of control or GSDME-overexpression HCT-116 and SW480 cells were determined by wounding-healing assay. **E** The MVD was positively correlated with GSDME expression in CRC tissue. All data were presented as the mean ± SD from three independent experiments. *P < 0.05, ** P < 0.01, ***P < 0.001

To examine the role of GSDME in cell migration and invasion, we stably expressed GSDME in HCT116 and SW480 cells (Fig. 7A). The transwell assays revealed that HCT116-GSDME and SW480-GSDME group exhibited more invasive (SW480: t = 4.218, p = 0.0135; HCT116: t = 9.627, p = 0.0007) and migrating (SW480: t = 5.003, p = 0.0075; HCT116: t = 6.354, p = 0.0031) than the control group (Fig. 7B, C). The wound-healing assay indicated that over expression of GSDME in HCT116 (24 h: t = 4.312, p = 0.0125; 48 h: t = 6.394, p = 0.0031, 72 h: t = 15.24, p = 0.0001) and SW480 cells (24 h: t = 3.726, p = 0.02; 48 h: t = 4.141, p = 0.014, 72 h: t = 12.55, p = 0.0002) increased the migratory rate (Fig. 7D).

### GSDME was involved in angiogenesis in colorectal cancer (CRC)

During the drug sensitivity analysis, we found that the some GSDME related drugs were angiogenesis inhibitors, such as Telatinib (r = 0.61) and Lenvatinib (r = 0.45) (Additional file 8: Data S1). In addition, during the tumor microenvironment estimation, we found that expression of GSDME was positively correlated with StromalScore in CRC(r = 0.84) (Additional file 11: Data S4). Based on this, we suspected that GSDME might related to angiogenesis in CRC tissue. Therefore, further investigation was taken into the correlation between expression of GSDME and endothelial cell (EC) markers using GEPIA [23]. As a result, the mRNA level of all the EC markers showed positively correlation with the mRNA level of GSDME in CRC tissue (Fig. 6B, Additional file 5: Fig. S5A). Gene Set Enrichment Analysis (GSEA) [24, 25] highlighted that the GO Endothelium Development Gene Set upregulation was related to increased expression of GSDME (Fig. 6C).

We doubted whether GSDME promoted the expression of vascular endothelial growth factor (VEGF) in CRC cells. We next analyzed the association between expression of GSDME and the VEGF in Cancer Cell Line Encyclopedia (CCLE). As expected, the expression of GSDME was positively correlated with VEGFB (r = 0.46) and VEGFC (r = 0.40) (Additional file 6: Fig. S6). Hence, we suspected that GSDME was positively correlated with the production of VEGF by cancer cell. Also, the expression level of GSDME associated (Fvalue = 2.913, Pr = 0.034) with stages of CRC (Fig. 6D). And as a contrast, we also analyzed GSDMC and found a positive correlation between the mRNA level of GSDMC and mRNA level of EC marker, but no significant correlation has been found between GSDMC and VEGF in CCLE (Additional file 7: Fig. S7). Since VEGF promoted cancer angiogenesis and metastasis [26], we suspected that GSDME might be involved in CRC growth and metastasis via VEGF related pathways in CRC.

To determine the expression of GSDME and angiogenesis, IHC assay was performed using 16 CRC tissue (Fig. 7E). The number of micro vessels per unit area (micro vessel density, MVD) was visualized by CD34 and revealed that angiogenesis was promoted in higher GSDME expression CRC tissue (t = 2.278, p = 0.039).

## Discussion

Since its first discovery more than two decades ago, a great amount of effort has been made to investigate the biofunction of GSDM family genes. Originally, GSDM genes were identified as candidate causative genes of hair loss and hearing loss [27–30]. The real breakthrough was that GSDMD was identified as the key executor of inflammatory cell death known as pyroptosis [5, 9, 31, 32], a lytic type of cell death associated with inflammation. Because of their similar architecture, we tend to believe all GSDM genes have the ability somehow to form pores in plasm or mitochondrial membrane and to trigger cell death following N-terminal activation. Therefore, most studies have focused on which site of GSDM gene has been cleaved by which enzymes, the caspase or other, thus activating the specific GSDM gene, forming pores in cells subsequently, triggering lytic cell death. Later, GSDME and GSDMB have been uncovered to play a critical role in inducing pyroptosis [11, 27], further reinforcing the idea that GSDM genes have the pore-forming and cell lysis ability. To date, however, there is little known about GSDMA, GSDMC and PJVK, although their N-terminal moieties generated by genetical engineering in vitro showed pore-forming activity that binds to membrane inner leaflet lipids and causes cell membrane permeabilization and pyroptosis [8, 33]. In addition, GSDM genes have been implicated in regulation of cancer behaviors, but whether it suppresses cancer or promotes cancer is controversial [34–37].

In this pan-cancer study, we found that (1) genome of GSDM genes were extensively altered in cancer and the corresponding patients showed survival disadvantage; (2) the expression of GSDM genes was altered in cancer due to genetic alteration and epigenetic modification of GSDM genome, and it affected patient survival; (3) GSDM family members were involved in cancer-related pathways in varying degrees and had a role in drug sensitivity; (4) the expression of GSDM genes associated with immune subtypes, TME and cell stemness of cancer. Our finding indicated that GSDM genes had an extensive involvement in cancer, more efforts should be made to elucidate the detailed mechanisms. However, we also discovered an intra- and inter-cancer heterogeneity regarding the corresponding genes. We suggest each GSDM gene be studied as an entity in each type of cancer.

Other than triggering pyroptosis, we suspected GSDM genes might play other roles in regulation of cancer cell behaviors. Though our analysis, we found that GSDMA, GSDMB, GSDMD and GSDME might be involved in colorectal cancer cell invasion and metastasis, and the former three might played as negative regulators while the last one played as a positive regulator. The expression of GSDME strongly positive correlation with stromal cells infiltration and the expression of EC markers in solid tissue of CRC. In CCLE, we found the expression of GSDME was significantly positively correlated with the expression of VEGFB and VEGFC in CRC cell lines. Thus, we suspected there was a crosstalk between GSDME and VEGF within the cancer cell and another crosstalk between cancer cell and TME, which was considered to play an important role in cancer progression, metastasis as well as drug resistance [38–42]. Also, our experiment verified that overexpression GSDME promoted the migration and invasion of SW480 and HCT116 cell lines and the positive correlation between GSDME expression and MVD. Upregulated GSDME by reversal of epigenetic silencing and facilitated the occurrence of pyroptosis was considered as an important pyroptosis-based cancer chemotherapy strategy [43], but we suggest more attention should be paid to the oncogenic role of GSMDE when being upregulated.

There were some limitations in our study. Different online databases usage might cause background heterogeneity. Besides, we didn’t carry out enough experiments to verify our findings. Next, more studies in vitro and in vivo will be carried out to verify the biological information results.

## Conclusion

GSDM family genes might play important roles in cancer. We suggest more efforts be made to investigate the GSDM family and each GSDM gene be studied as an entity in each type of cancer. While studying pyroptosis, other roles GSDM genes might play should not be ignored. And, GSDMA, GSDMB and GSDMD exhibited a lot of differences, even opposite aspects to GSDME, and we hope this might provide some reference for the future research.

## Supplementary Information

**Additional file 1: Figure S1.** Phylogenetic relationship, gene structure, and conserved motif analysis of the GSDM family genes (Mus muculus and Homo sapiens). (A) Phylogenetic analysis of GSDM family genes. (B) Gene structure analysis of GSDM family genes. (C) Conserved motifs discovered by MEME.

**Additional file 2: Figure S2.** The protein domain structure and activating and inhibitory cleavage sites of GSDM family genes. All GSDM genes but PJVK have a pore-forming Gasdermin domain and a regulatory C-terminal domain. Cleavage at the sites marked by a red rectangle is considered to be prerequisite for activation, while cleavage at the sites marked by a blue rectangle generates nontoxic N-terminal moieties.

**Additional file 3: Figure S3.** Correlation plots for GSDM gene copy number alterations and corresponding mRNA expression in pan-cancer.

**Additional file 4: Figure S4**. Correlation between patient survival and immune subtypes. Patients classified into type C3 and C5 associated with significant survival advantage while patients characterized into type C4 and C6 had a significant survival disadvantage during ten years' follow-up.

**Additional file 5: Figure S5.** Matrix graph of Spearman's Rank-Order Correlation between GSDM genes expression and Estimatescore.

**Additional file 6: Figure S6.** GSDME was involved in angiogenesis in colorectal cancer. (A)GSDME expression positively correlated several EC markers based on TCGA-COAD dataset analysis. (B) Expression of GSDME was positively correlated with endogenous expression of VEGF in colorectal cell lines based on Broad Institute Cancer Cell Line Encyclopedia (CCLE) dataset analysis.

**Additional file 7: Figure S7.** Correlation between the mRNA level of GSDMC and mRNA level of EC markers. (A) GSDMC expression positively correlated EC markers. (B) Increased expression of GSDMC provided endothelial development advantage based on Gene Set Enrichment Analysis (GSEA). (C-D) Expression of GSDMC was NOT correlated with endogenous expression of VEGF in colorectal cell lines based on Broad Institute Cancer Cell Line Encyclopedia (CCLE) dataset analysis.

**Additional file 8: Data S1.** The correlation between the expression of GSDM family genes and drug sensitivity.

**Additional file 9: Data S2.** The correlation coeffecient for the Heatmap in Figure 3B.

**Additional file 10: Data S3.** The univariate Cox proportional hazards model for correlation between GSDM genes expression and patient survival.

**Additional file 11: Data S4.** The correlation between the expression of GSDM genes and ImmuneScore/StromalScore/EstimateScore.

**Additional file 12: Data S5.** The ImmuneScore/StromalScore/EstimateScore and TumorPurity of TCGA patients.

**Additional file 13: Data S6.** The correlation between the expression of GSDM genes and DNAss/RNAss.

## Data Availability

Most of the data in this study are included either in this article or in the Additional files, other are available from the corresponding author on reasonable request.

## Abbreviations

GSDMs: Gasdermins
TCGA: The Cancer Genome Atlas
CCLE: Cancer Cell Line Encyclopedia
GEPIA: Gene Expression Profiling Interactive Analysis
DMEM: Dulbecco’s modified Eagle’s medium
FBS: Fetal bovine serum
CANs: Copy number alterations
EMT: Epithelial Mesenchymal Transition
PPI: Protein–protein-interaction
PFS: Progression free survival
OS: Overall survival
ISs: Immune subtypes
MVD: Micro vessel density
