# Supplementary figures and images for: A pan-cancer analysis of molecular characteristics and oncogenic role of gasdermins
Source: Cancer Cell Int. 2022 Feb 14;22:80. doi: 10.1186/s12935-022-02483-4 (PMC8842873; doi:10.1186/s12935-022-02483-4)

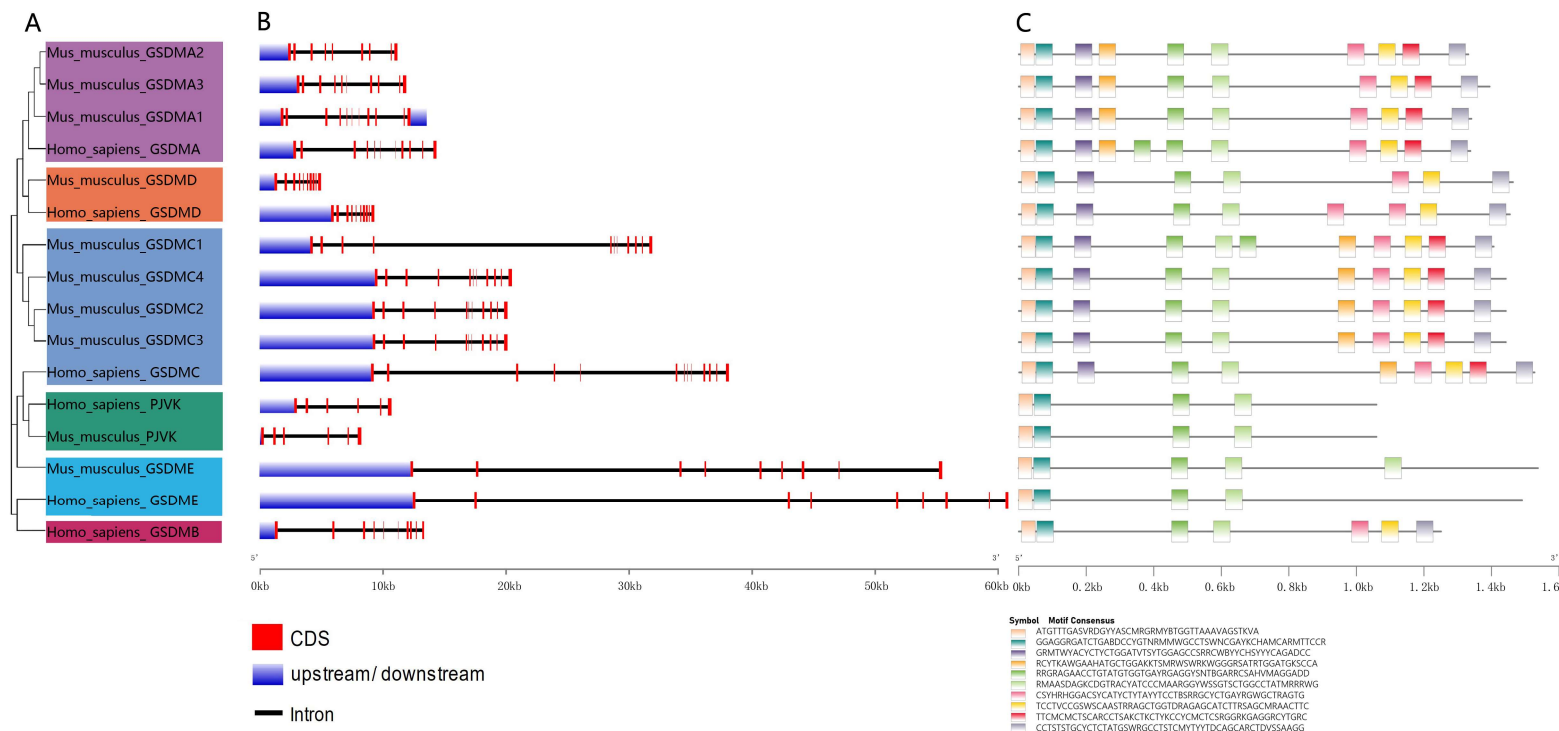

Supplement: Supplementary file 1 — Additional file 1: Figure S1. Phylogenetic relationship, gene structure, and conserved motif analysis of the GSDM family genes (Mus muculus and Homo sapiens). (A) Phylogenetic analysis of GSDM family genes. (B) Gene structure analysis of GSDM family genes. (C) Conserved motifs discovered by MEME. [file 12935_2022_2483_MOESM1_ESM.pdf]

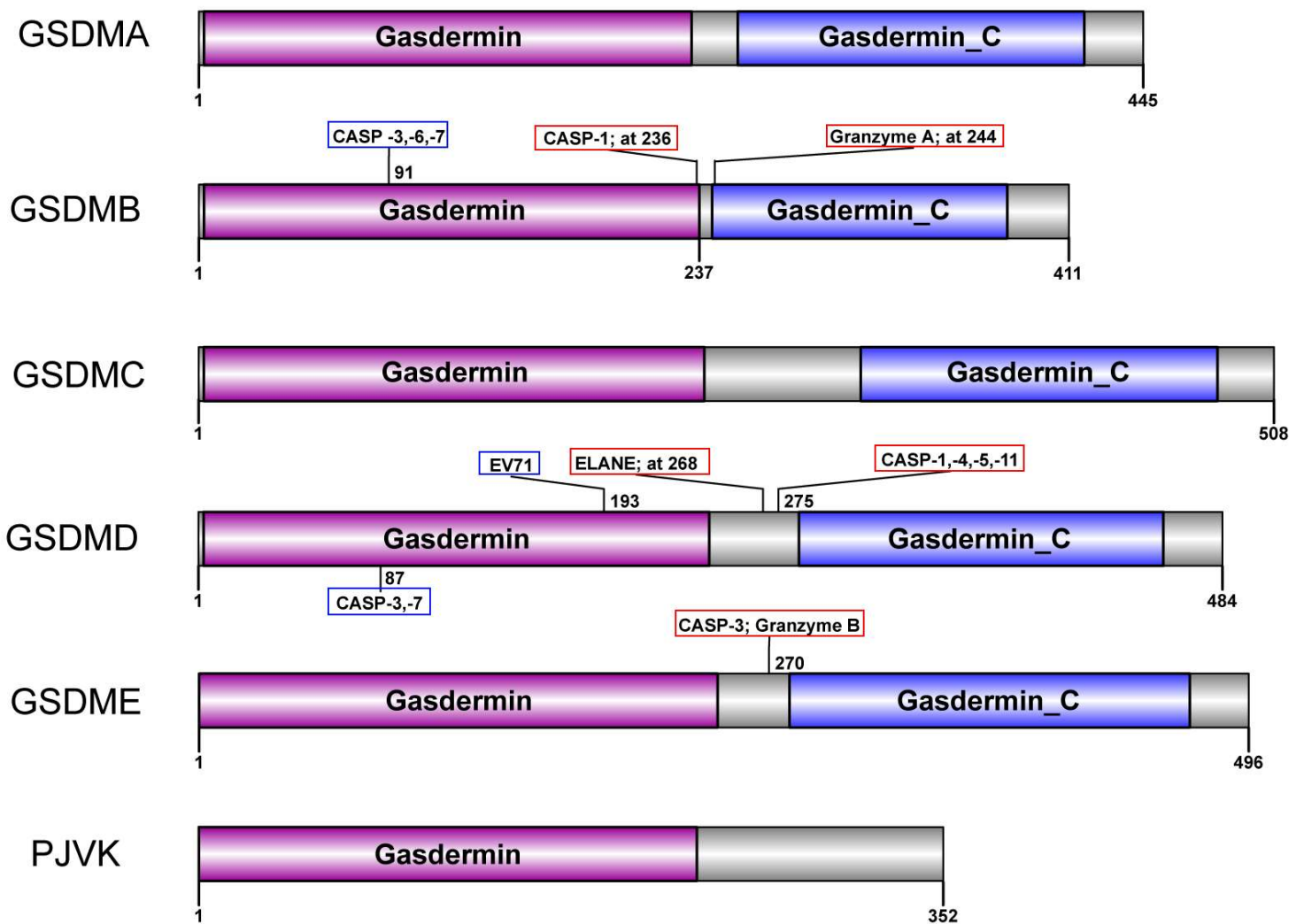

Supplement: Supplementary file 2 — Additional file 2: Figure S2. The protein domain structure and activating and inhibitory cleavage sites of GSDM family genes. All GSDM genes but PJVK have a pore-forming Gasdermin domain and a regulatory C-terminal domain. Cleavage at the sites marked by a red rectangle is considered to be prerequisite for activation, while cleavage at the sites marked by a blue rectangle generates nontoxic N-terminal moieties. [file 12935_2022_2483_MOESM2_ESM.pdf]

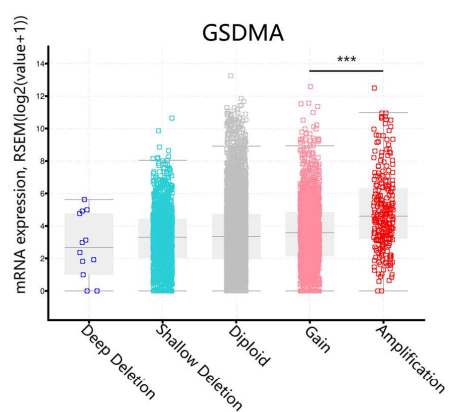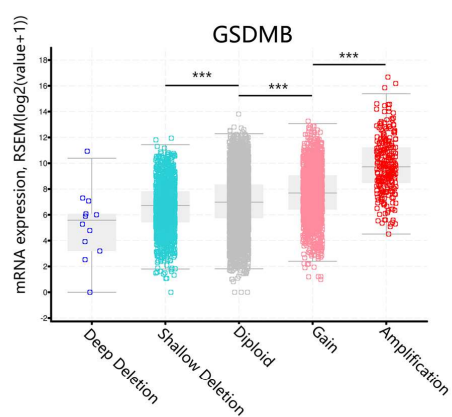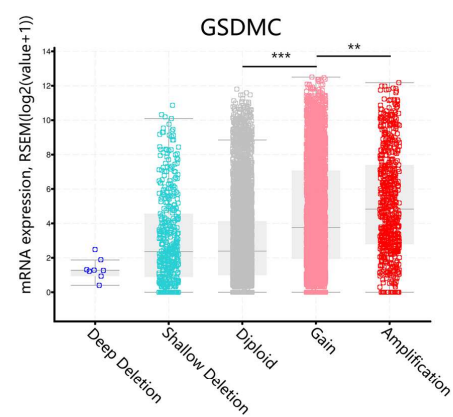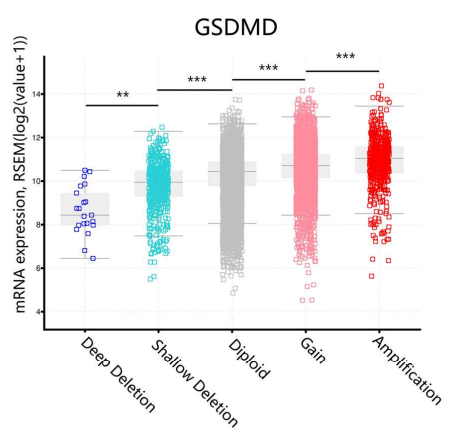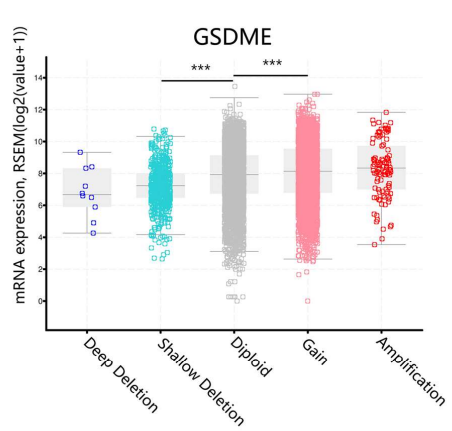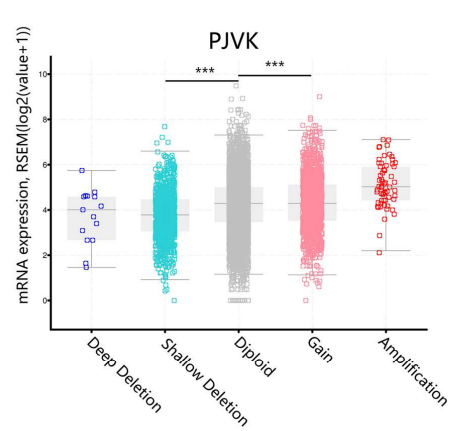

■ Amplification 
 ■ Gain 
 ■ Diploid 
 ■ Shallow Deletion 
 ■ Deep Deletion 
 \*\* P<0.05 
 \*\*\*P<0.01

Supplement: Supplementary file 3 — Additional file 3: Figure S3. Correlation plots for GSDM gene copy number alterations and corresponding mRNA expression in pan-cancer. [file 12935_2022_2483_MOESM3_ESM.pdf]

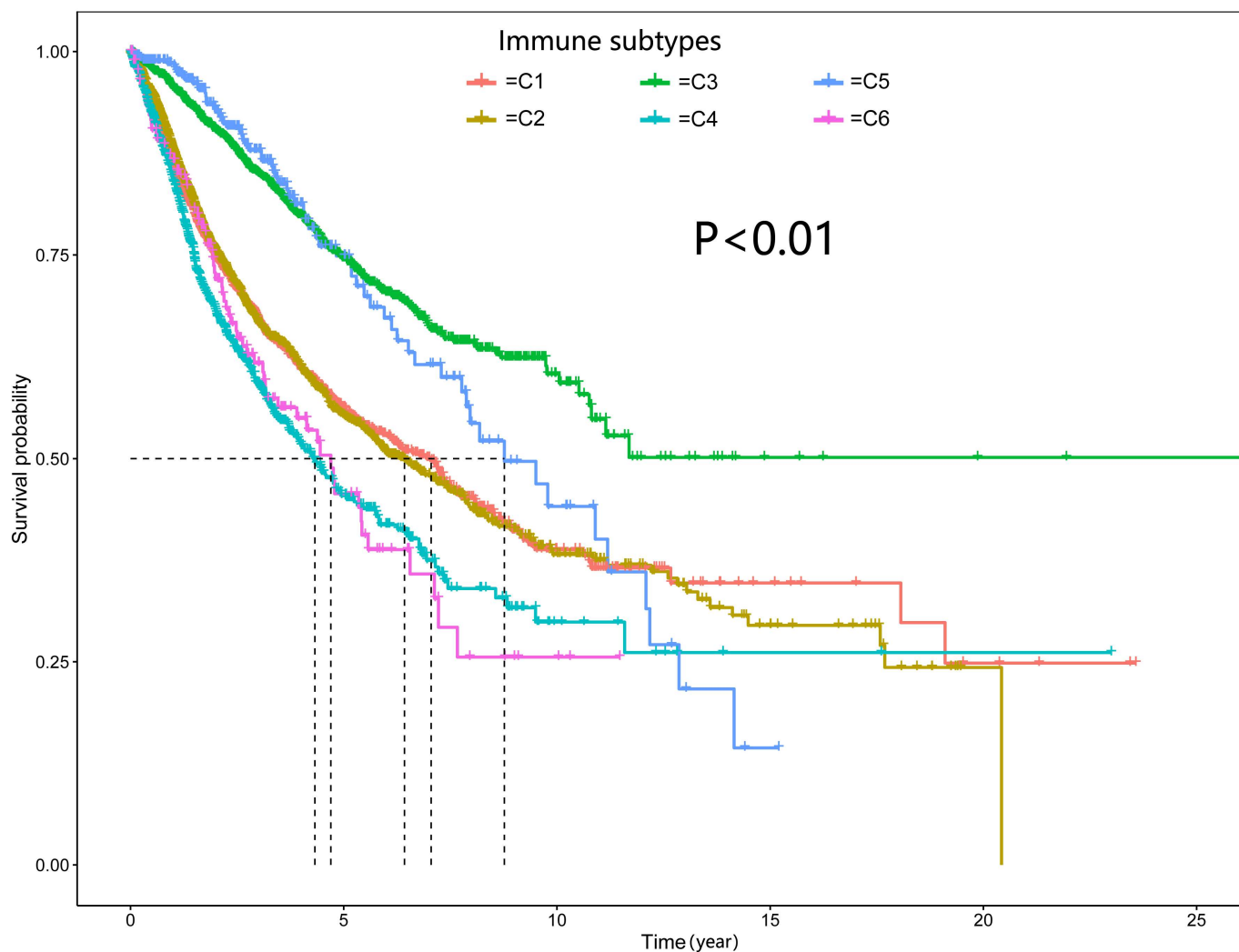

**Number at risk**

|    |      |     |    |    |   |   |
|----|------|-----|----|----|---|---|
| C1 | 2307 | 306 | 56 | 11 | 4 | 0 |
| C2 | 2459 | 394 | 77 | 23 | 1 | 0 |
| C3 | 2364 | 485 | 55 | 5  | 2 | 1 |
| C4 | 1132 | 123 | 11 | 2  | 1 | 0 |
| C5 | 376  | 64  | 15 | 1  | 0 | 0 |
| C6 | 175  | 29  | 3  | 0  | 0 | 0 |

Time (year)

Supplement: Supplementary file 4 — Additional file 4: Figure S4. Correlation between patient survival and immune subtypes. Patients classified into type C3 and C5 associated with significant survival advantage while patients characterized into type C4 and C6 had a significant survival disadvantage during ten years' follow-up. [file 12935_2022_2483_MOESM4_ESM.pdf]

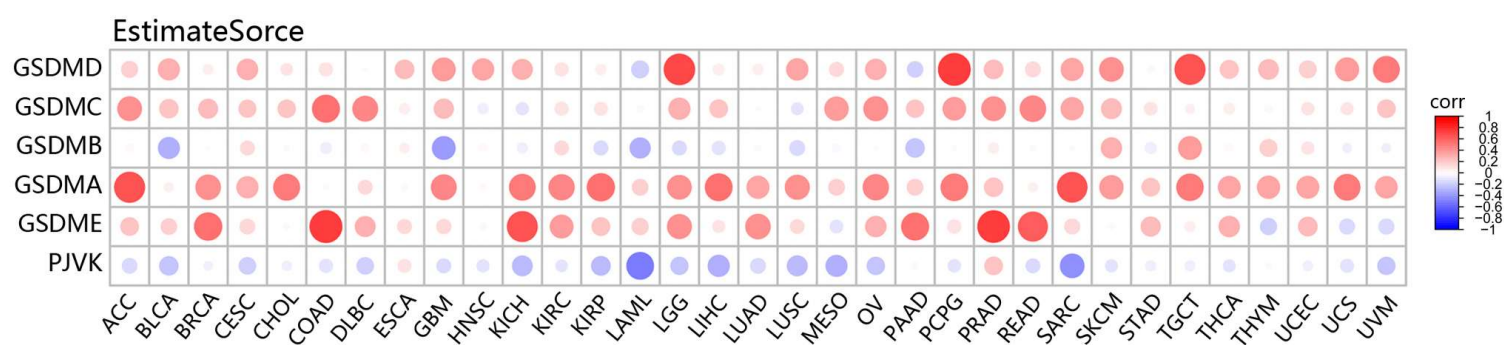

Supplement: Supplementary file 5 — Additional file 5: Figure S5. Matrix graph of Spearman's Rank-Order Correlation between GSDM genes expression and Estimatescore. [file 12935_2022_2483_MOESM5_ESM.pdf]

**A**

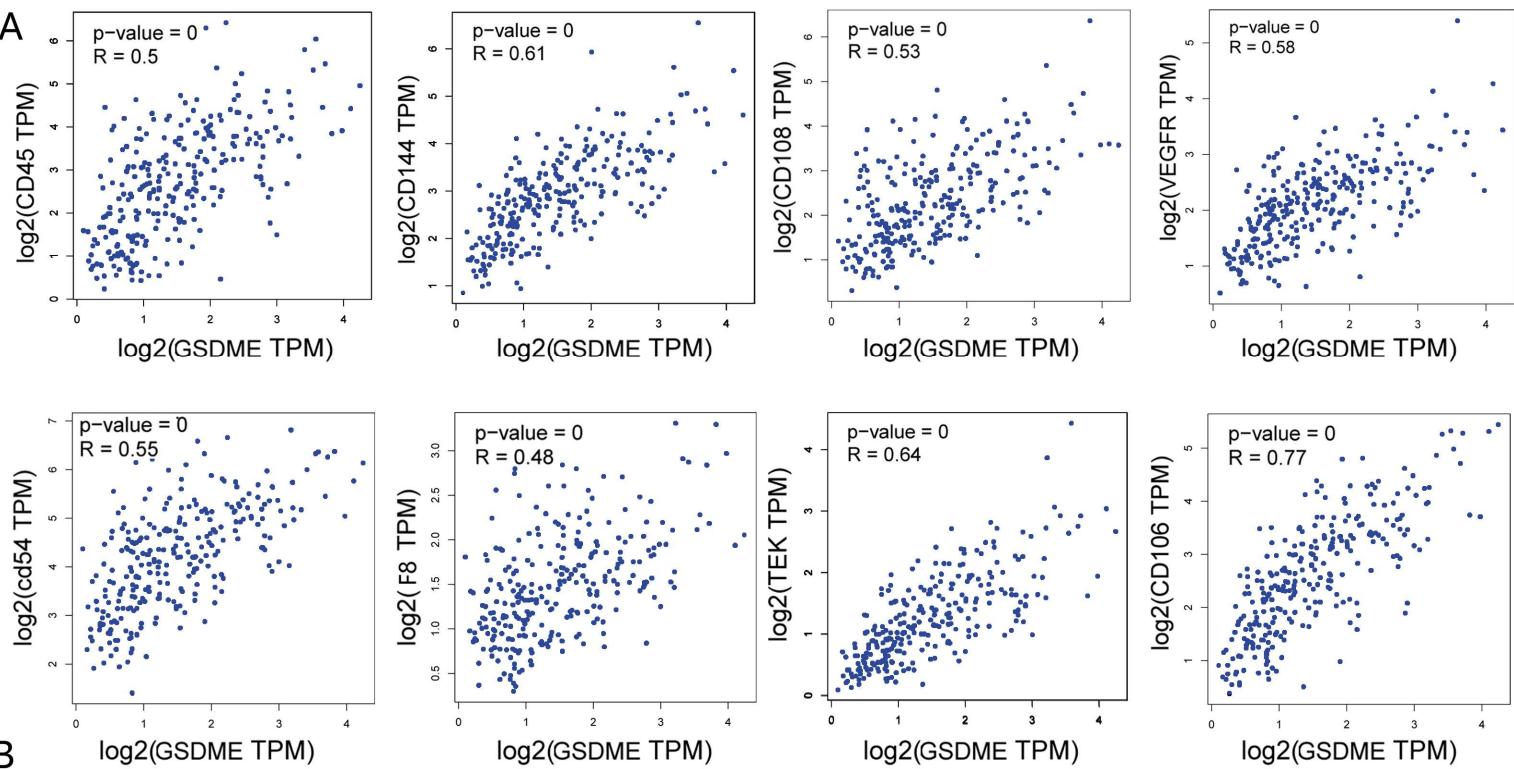

**B**

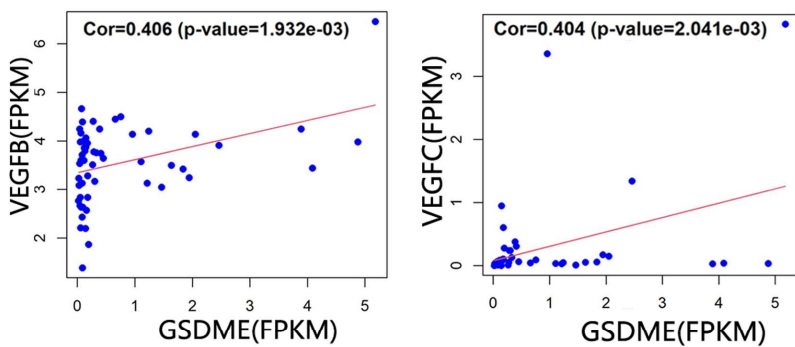

Supplement: Supplementary file 6 — Additional file 6: Figure S6. GSDME was involved in angiogenesis in colorectal cancer. (A)GSDME expression positively correlated several EC markers based on TCGA-COAD dataset analysis. (B) Expression of GSDME was positively correlated with endogenous expression of VEGF in colorectal cell lines based on Broad Institute Cancer Cell Line Encyclopedia (CCLE) dataset analysis. [file 12935_2022_2483_MOESM6_ESM.pdf]

A

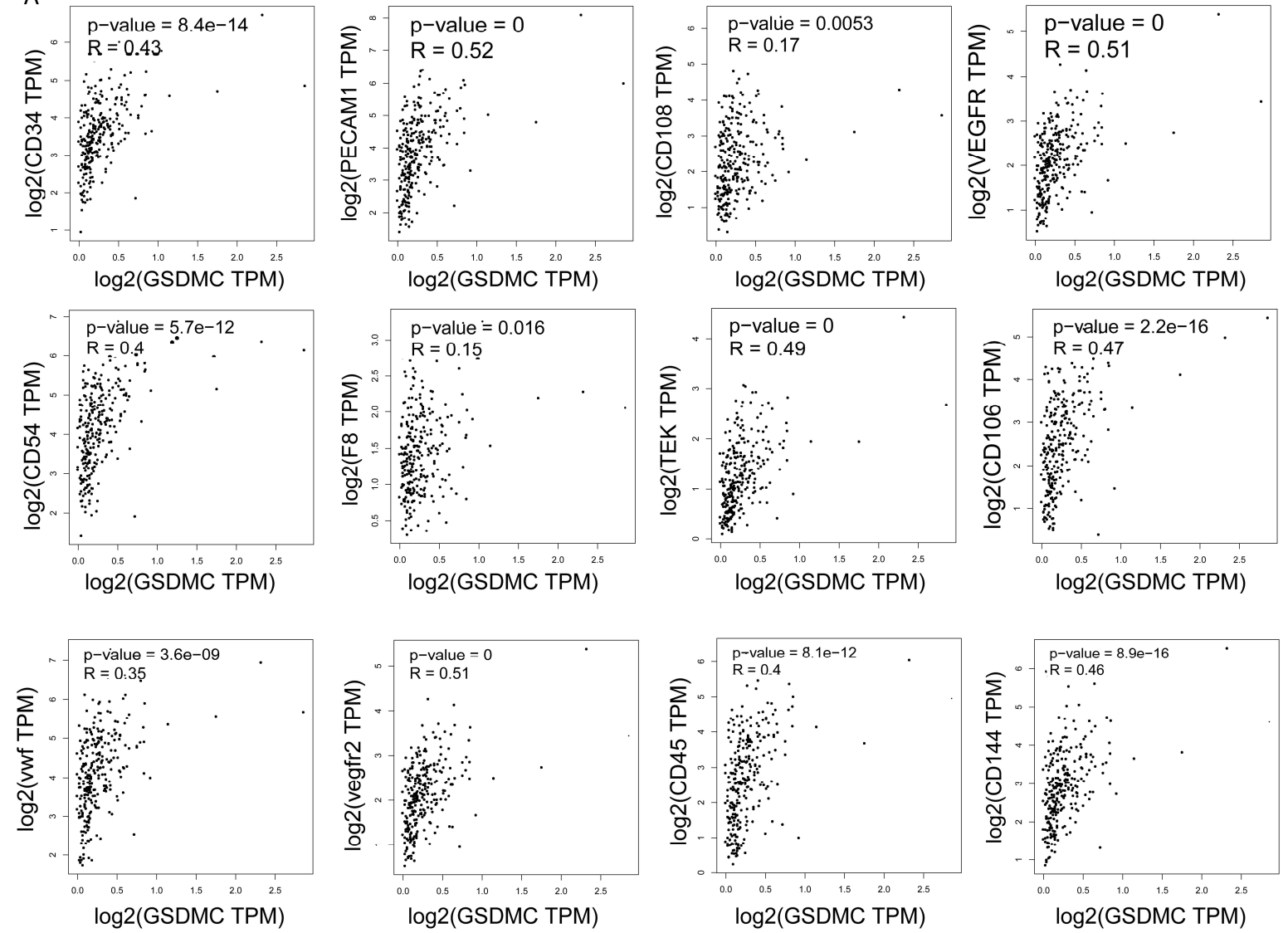

B

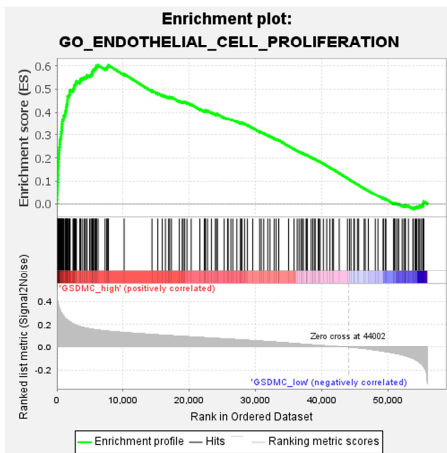

C

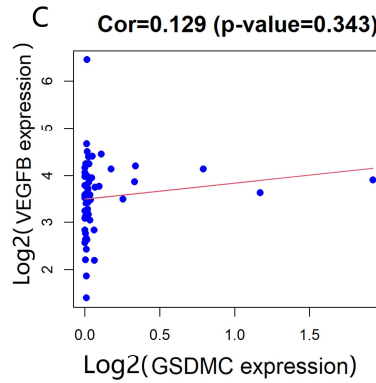

D

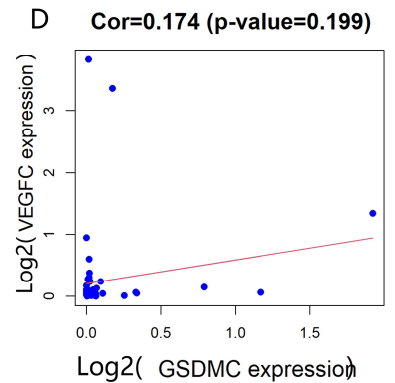

Supplement: Supplementary file 7 — Additional file 7: Figure S7. Correlation between the mRNA level of GSDMC and mRNA level of EC markers. (A) GSDMC expression positively correlated EC markers. (B) Increased expression of GSDMC provided endothelial development advantage based on Gene Set Enrichment Analysis (GSEA). (C-D) Expression of GSDMC was NOT correlated with endogenous expression of VEGF in colorectal cell lines based on Broad Institute Cancer Cell Line Encyclopedia (CCLE) dataset analysis. [file 12935_2022_2483_MOESM7_ESM.pdf]
